# Supplementary figures and images for: DNA barcoding of brackish and marine water fishes and shellfishes of Sundarbans, the world’s largest mangrove ecosystem
Source: PLoS One. 2021 Aug 2;16(8):e0255110. doi: 10.1371/journal.pone.0255110 (PMC8328341; doi:10.1371/journal.pone.0255110)

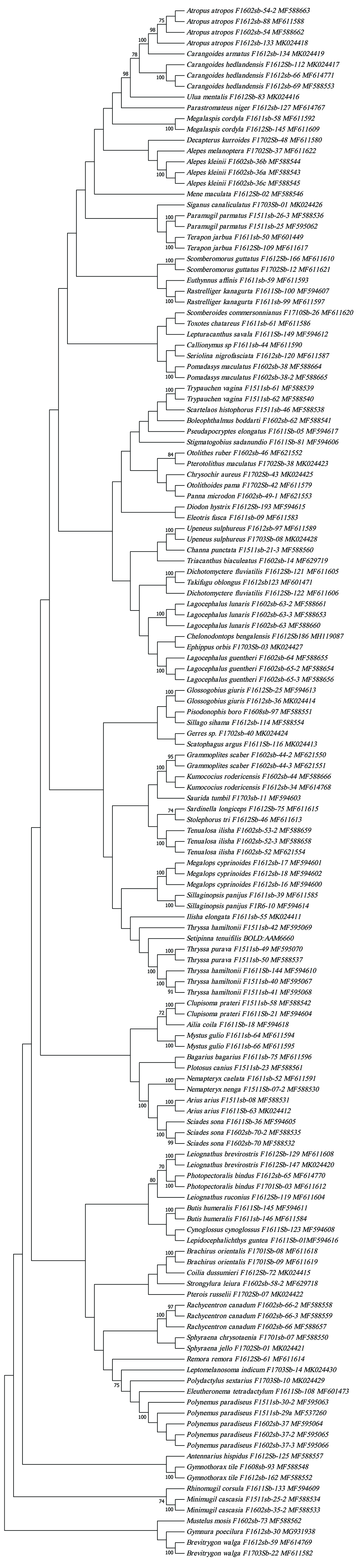

Supplement: S1 Fig — Bootstrap support of ≥70% are shown above branches. Scale represents genetic distance between species. (TIF) [file pone.0255110.s001.tif]

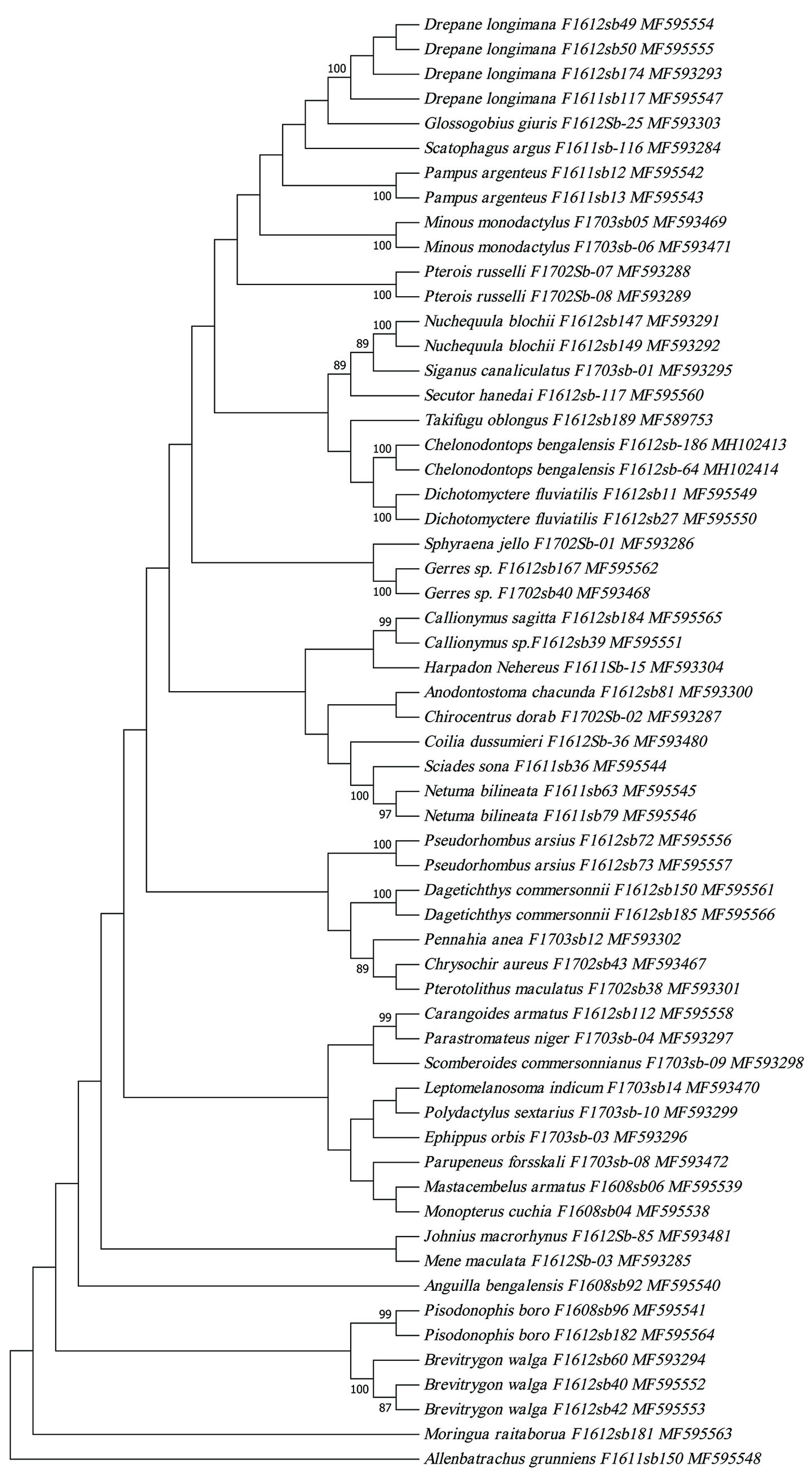

Supplement: S2 Fig — Bootstrap support of >70% are shown above branches. Scale represents genetic distance between species. Bootstrap replications 1000. (TIF) [file pone.0255110.s002.tif]

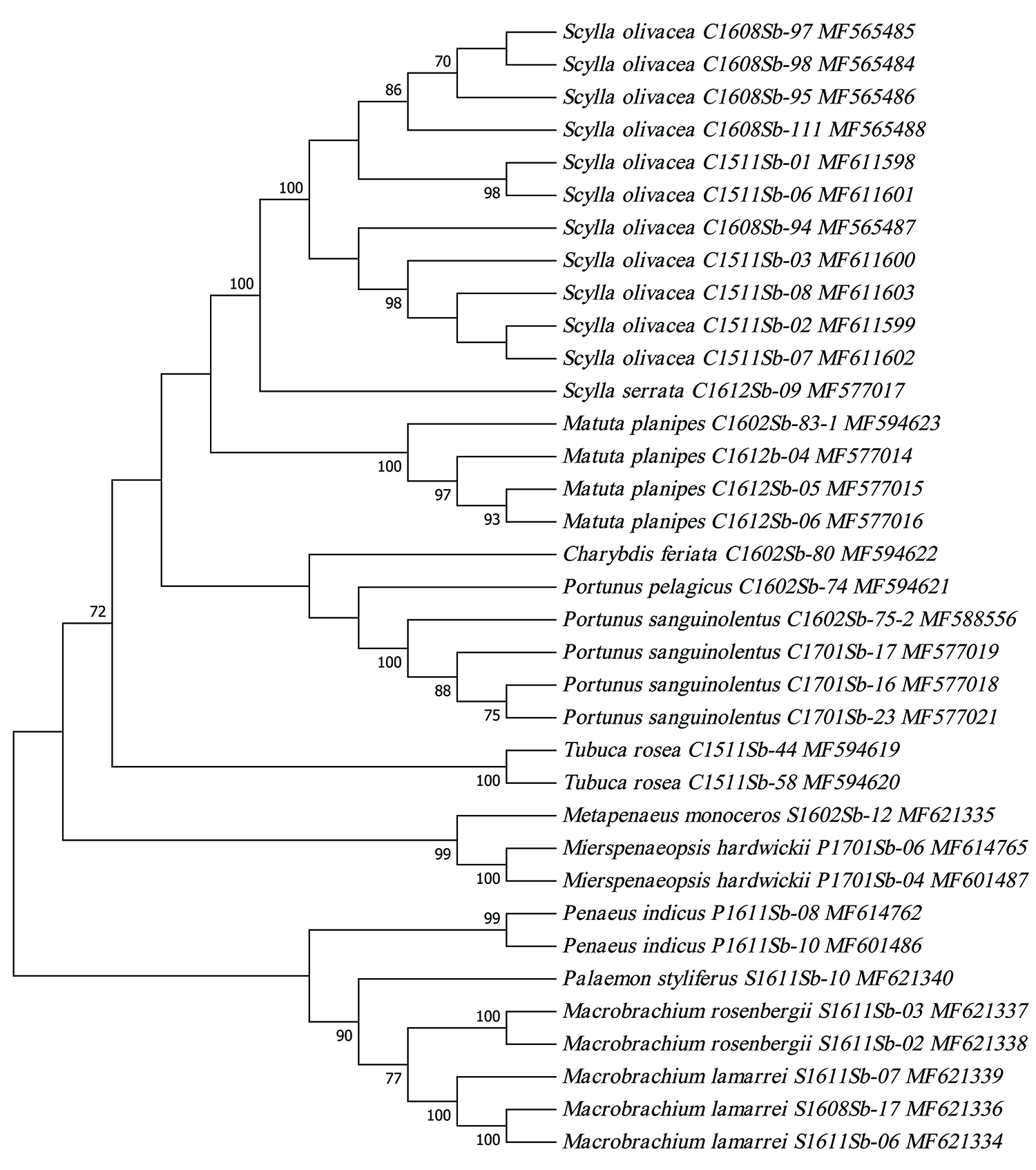

Supplement: S3 Fig — Bootstrap support of >70% are shown above branches. Scale represents genetic distance between species. Bootstrap replications 10000. (TIF) [file pone.0255110.s003.tif]

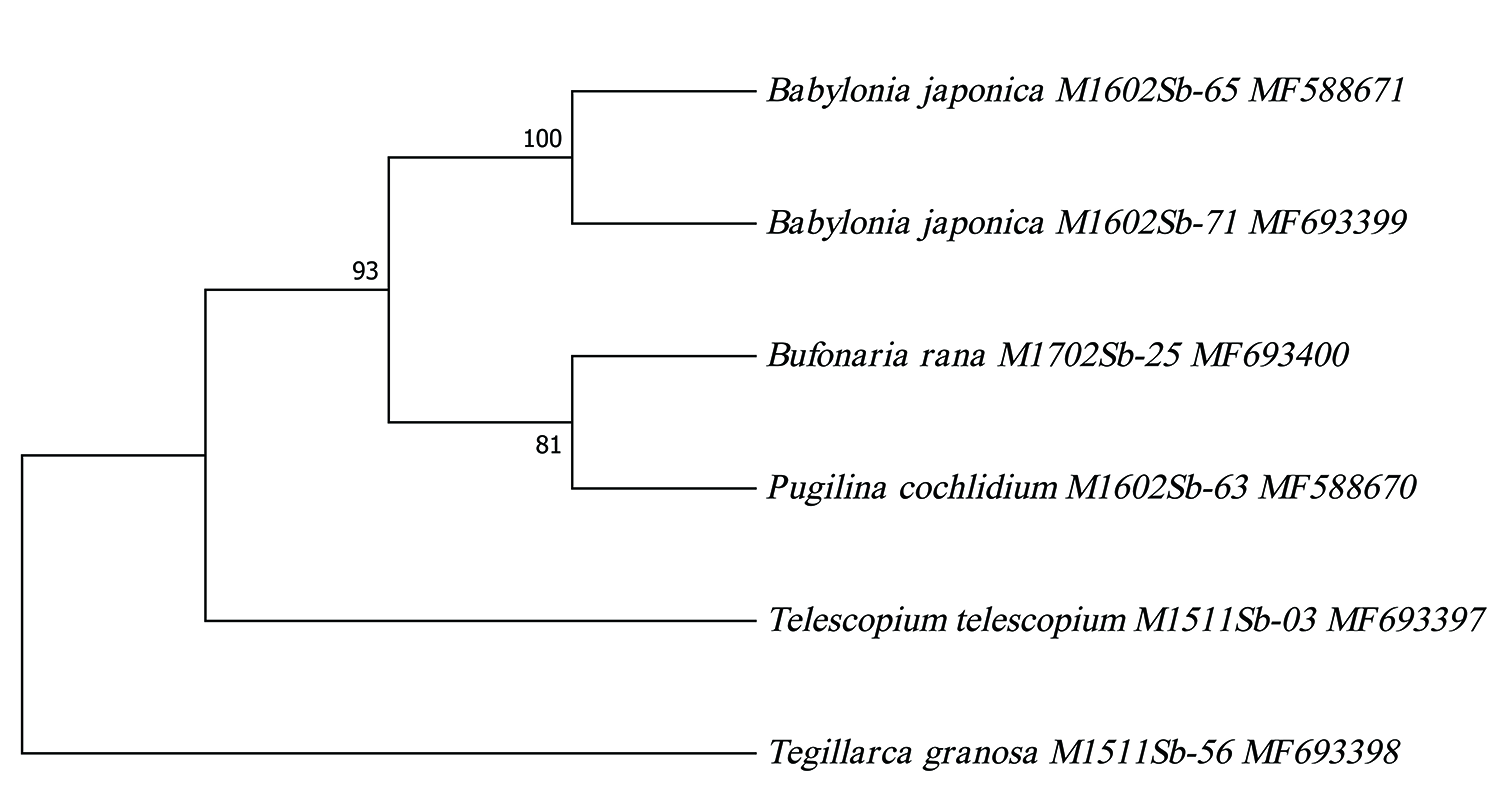

Supplement: S4 Fig — Bootstrap support of >70% are shown above branches. Scale represents genetic distance between species. Bootstrap replications 10000. (TIF) [file pone.0255110.s004.tif]

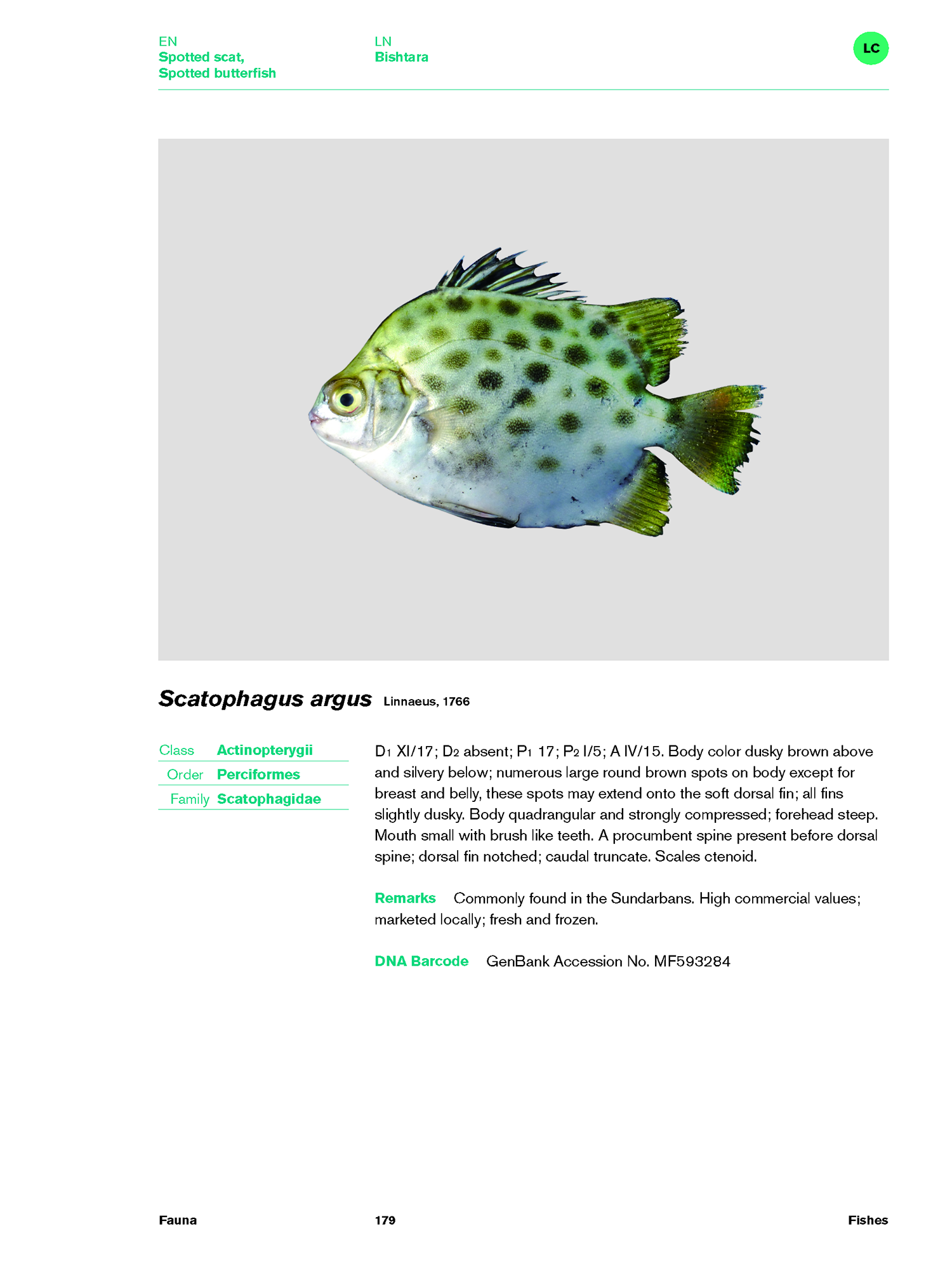

Supplement: S5 Fig — (TIF) [file pone.0255110.s005.tif]
