# Supplementary material for: DNA barcoding of brackish and marine water fishes and shellfishes of Sundarbans, the world’s largest mangrove ecosystem
Source: PLoS One. 2021 Aug 2;16(8):e0255110. doi: 10.1371/journal.pone.0255110 (PMC8328341; doi:10.1371/journal.pone.0255110)
Supplement: S1 Table — (PDF) [file pone.0255110.s006.pdf]

S1 Table

Order	Family	Scientific Name	COI	16S
Fishes				
Anguilliformes	Anguillidae	*Anguilla bengalensis*		MF595540
Anguilliformes	Muraenidae	*Gymnothorax tile*	MF588548, MF588552	
Anguilliformes	Anguillidae	*Moringua raitaborua*		MF595563
Anguilliformes	Ophichthidae	*Pisodonophis boro*	MF588551	MF595541, MF595564
Aulopiformes	Synodontidae	*Harpadon nehereus*		MF593304
Aulopiformes	Synodontidae	*Saurida tumbil*	MF594603	
Batrachoidiformes	Batrachoididae	*Allenbatrachus grunniens*		MF595548
Beloniformes	Belonidae	*Strongylura leiura*	MF629718	
Carcharhiniformes	Triakidae	*Mustelus mosis*	MF588562	
Clupeiformes	Clupeidae	*Anodontostoma chacunda*		MF593300
Clupeiformes	Chirocentridae	*Chirocentrus dorab*		MF593287
Clupeiformes	Engraulidae	*Coilia dussumieri*	MK024415	MF593480
Clupeiformes	Pristigasteridae	*Ilisha elongata*	MK024411	
Clupeiformes	Clupeidae	*Sardinella longiceps*	MF611615	
Clupeiformes	Engraulidae	*Setipinna tenuifilis*	MF611616	
Clupeiformes	Engraulidae	*Stolephorus tri*	MF611613	
Clupeiformes	Clupeidae	*Tenualosa ilisha*	MF588659, MF588658, MF621554	
Clupeiformes	Engraulidae	*Thryssa hamiltonii*	MF595067, MF595068, MF594610	
Clupeiformes	Engraulidae	*Thryssa purava*	MF588537, MF595070	
Cypriniformes	Cobitidae	*Lepidocephalichthys guntea*	MF594616	
Elopiformes	Megalopidae	*Megalops cyprinoides*	MF594600, MF594601, MF594602	
Lophiiformes	Antennariidae	*Antennarius hispidus*	MF588557	
Myliobatiformes	Dasyatidae	*Brevitrygon walga*	MF611582, MF614769	MF593294, MF595552, MF595553
Myliobatiformes	Gymnuridae	*Gymnura poecilura*	MG931938	
Perciformes	Carangidae	*Alepes kleinii*	MF588543, MF588544, MF588545	
Perciformes	Carangidae	*Alepes melanoptera*	MF611622	
Perciformes	Carangidae	*Atropus atropos*	MF588663, MF611588, MF588662, MK024418	

Order	Family	Scientific Name	COI	16S
Perciformes	Gobiidae	*Boleophthalmus boddarti*	MF588541	
Perciformes	Soleidae	*Brachirus orientalis*	MF611618, MF611619	
Perciformes	Eleotridae	*Butis humeralis*	MF611584, MF594611	
Perciformes	Callionymidae	*Callionymus sagitta*		MF595565
Perciformes	Callionymidae	*Callionymus sp.*	MF611590	MF595551
Perciformes	Carangidae	*Carangoides armatus*	MK024419	MF595558
Perciformes	Carangidae	*Carangoides hedlandensis*	MK024417, MF614771, MF588553	
Perciformes	Sciaenidae	*Chrysochir aureus*	MK024425	MF593467
Perciformes	Cynoglossidae	*Cynoglossus cynoglossus*	MF594608	
Perciformes	Soleidae	*Dagetichthys commersonnii*		MF595561, MF595566
Perciformes	Carangidae	*Decapterus kurroides*	MF611580	
Perciformes	Leiognathidae	*Secutor hanedai*		MF595560
Perciformes	Drepanidae	*Drepane longimana*		MF595554, MF595555, MF593293, MF595547
Perciformes	Eleotridae	*Eleotris fusca*	MF611583	
Perciformes	Polynemidae	*Eleutheronema tetradactylum*	MF601473	
Perciformes	Drepanidae	*Ephippus orbis*	MK024427	MF593296
Perciformes	Scombridae	*Euthynnus affinis*	MF611593	
Perciformes	Gerreidae	*Gerres sp.*	MK024424	MF595562, MF593468
Perciformes	Gobiidae	*Glossogobius giuris*	MF594613, MK024414	MF593303
Perciformes	Sciaenidae	*Johnius macrorhynus*		MF593481
Perciformes	Leiognathidae	*Leioghnathus brevirostris*	MF611608, MK024420	
Perciformes	Polynemidae	*Leptomelanosoma indicum*	MK024430	MF593470
Perciformes	Trichiuridae	*Lepturacanthus savala*	MF594612	
Perciformes	Carangidae	*Megalaspis cordyla*	MF611592, MF611609	
Perciformes	Menidae	*Mene maculata*	MF588546	MF593285
Perciformes	Mugilidae	*Minimugil cascasia*	MF588533, MF588534	
Perciformes	Leiognathidae	*Nuchequula blochii*		MF593291, MF593292
Perciformes	Sciaenidae	*Otolithes ruber*	MF621552	
Perciformes	Sciaenidae	*Otolithoides pama*	MF611579	
Perciformes	Stromateidae	*Pampus argenteus*		MF595542, MF595543
Perciformes	Sciaenidae	*Panna microdon*	MF621553	

Order	Family	Scientific Name	COI	16S
Perciformes	Mugilidae	*Paramugil parmatus*	MF588536, MF595062	
Perciformes	Carangidae	*Parastromateus niger*	MF614767	MF593297
Perciformes	Mullidae	*Parupeneus forsskali*		MF593296
Perciformes	Sciaenidae	*Pennahia anea*		MF593302
Perciformes	Leiognathidae	*Photopectoralis bindus*	MF614770, MF611612	
Perciformes	Polynemidae	*Polydactylus sextarius*	MK024429	MF593299
Perciformes	Polynemidae	*Polynemus paradiseus*	MF595063, MF537260, MF595064, MF595065, MF595066	
Perciformes	Haemulidae	*Pomadasys maculatus*	MF588664, MF588665	
Perciformes	Gobiidae	*Pseudapocryptes elongatus*	MF594617	
Perciformes	Paralichthyidae	*Pseudorhombus arsius*		MF595556, MF595557
Perciformes	Sciaenidae	*Pterotolithus maculatus*	MK024423	MF593301
Perciformes	Rachycentridae	*Rachycentron canadum*	MF588557, MF588558, MF588559	
Perciformes	Scombridae	*Rastrelliger kanagurta*	MF594607, MF611597	
Perciformes	Echeneidae	*Remora remora*	MF611614	
Perciformes	Mugilidae	*Rhinomugil corsula*	MF594609	
Perciformes	Gobiidae	*Scartelaos histophorus*	MF588538	
Perciformes	Scatophagidae	*Scatophagus argus*	MK024413	MF593284
Perciformes	Carangidae	*Scomberoides commersonnianus*	MF611620	MF593298
Perciformes	Scombridae	*Scomberomorus guttatus*	MF611610, MF611621	
Perciformes	Leiognathidae	*Secutor ruconius*	MF611604	
Perciformes	Carangidae	*Seriolina nigrofasciata*	MF611587	
Perciformes	Siganidae	*Siganus canaliculatus*	MK024426	MF593295
Perciformes	Sillaginidae	*Sillaginopsis panijus*	MF611585, MF594614	
Perciformes	Sillaginidae	*Sillago sihama*	MF588554	
Perciformes	Sphyraenidae	*Sphyraena chrysotaenia*	MF588550	
Perciformes	Sphyraenidae	*Sphyraena jello*	MK024421	MF593286
Perciformes	Gobiidae	*Stigmatogobius sadanundio*	MF594606	
Perciformes	Terapontidae	*Terapon jarbua*	MF601449, MF611617	
Perciformes	Toxotidae	*Toxotes chatareus*	MF611586	
Perciformes	Gobiidae	*Trypauchen vagina*	MF588539, MF588540	

Order	Family	Scientific Name	COI	16S
Perciformes	Carangidae	*Ulua mentalis*	MK024416	
Perciformes	Mullidae	*Upeneus sulphureus*	MK024428, MF611589	
Scorpaeniformes	Platycephalidae	*Grammoplites scaber*	MF621550, MF621551	
Scorpaeniformes	Synanceiidae	*Minous monodactylus*		MF593469, MF593471
Scorpaeniformes	Scorpaenidae	*Pterois russelii*	MK024422	MF593288, MF593289
Scorpaeniformes	Platycephaloidei	*Kumococius rodericensis*	MF588666, MF614768	
Siluriformes	Schilbeidae	*Ailia coila*	MF594618	
Siluriformes	Ariidae	*Arius arius*	MK024412, MF588531	
Siluriformes	Sisoridae	*Bagarius bagarius*	MF611596	
Siluriformes	Schilbeidae	*Clupisoma prateri*	MF588542, MF594604	
Siluriformes	Ariidae	*Sciades sona*	MF588532, MF594605, MF588535	MF595544
Siluriformes	Bagridae	*Mystus gulio*	MF611594, MF611595	
Siluriformes	Ariidae	*Nemapteryx caelata*	MF611591	
Siluriformes	Ariidae	*Nemapteryx nenga*	MF588530	
Siluriformes	Ariidae	*Netuma bilineata*		MF595545, MF595546
Siluriformes	Plotosidae	*Plotosus canius*	MF588561	
Synbranchiformes	Mastacembelidae	*Mastacembelus armatus*		MF595539
Synbranchiformes	Synbranchidae	*Monopterus cuchia*		MF595538
Tetraodontiformes	Tetraodontidae	*Chelonodontops bengalensis*	MH119087	MH102413, MH102414
Tetraodontiformes	Tetraodontidae	*Dichotomyctere fluviatilis*	MF611605, MF611606	MF595549, MF595550
Tetraodontiformes	Tetraodontidae	*Diodon hystrix*	MF594615	
Tetraodontiformes	Tetraodontidae	*Lagocephalus guentheri*	MF588654, MF588655, MF588656	
Tetraodontiformes	Tetraodontidae	*Lagocephalus lunaris*	MF588661, MF588653, MF588660	
Tetraodontiformes	Tetraodontidae	*Takifugu oblongus*	MF601471	MF589753
Tetraodontiformes	Triacanthidae	*Triacanthus biaculeatus*	MF629719	
Crustaceans				
Decapoda	Portunidae	*Charybdis feriata*	MF594622	
Decapoda	Portunidae	*Portunus pelagicus*	MF594621	
Decapoda	Portunidae	*Portunus sanguinolentus*	MF577018, MF588556,	

Order	Family	Scientific Name	COI	16S
			MF577019, MF577021	
Decapoda	Portunidae	*Scylla olivacea*	MF611603, MF565486, MF611598, MF611599, MF611600, MF611601, MF611602, MF565488, MF565487, MF565485, MF565484	
Decapoda	Portunidae	*Scylla serrata*	MF577017	
Decapoda	Penaeidae	*Metapenaeus monoceros*	MF621335	
Decapoda	Penaeidae	*Penaeus indicus*	MF614762, MF601486	
Decapoda	Penaeidae	*Mierspenaeopsis hardwickii*	MF614765, MF601487	
Decapoda	Palaemonidae	*Palaemon styliferus*	MF621340	
Decapoda	Palaemonidae	*Macrobrachium rosenbergii*	MF621337, MF621338	
Decapoda	Palaemonidae	*Macrobrachium lamarrei*	MF621334, MF621339, MF621336	
Decapoda	Matutidae	*Matuta planipes*	MF594623, MF577014, MF577015, MF577016	
Decapoda	Ocypodidae	*Tubuca rosea*	MF594619, MF594620	
Molluscs				
Neogastropoda	Babyloniidae	*Babylonia japonica*	MF588671, MF693399	
Neogastropoda	Babyloniidae	*Bufonaria rana*	MF693400	
Neogastropoda	Melongenidae	*Volegalea cochlidium*	MF588670	
Caenogastropoda	Potamididae	*Telescopium telescopium*	MF693397	
Arcida	Arcidae	*Tegillarca granosa*	MF693398	
