# Supplementary material for: DNA barcoding of brackish and marine water fishes and shellfishes of Sundarbans, the world’s largest mangrove ecosystem
Source: PLoS One. 2021 Aug 2;16(8):e0255110. doi: 10.1371/journal.pone.0255110 (PMC8328341; doi:10.1371/journal.pone.0255110)
Supplement: Attachment — Submitted filename: Rebuttal letter.pdf [file pone.0255110.s008.pdf]

Dear Academic Editor,

Thank you so much for your emails. Here we have provided a detailed clarification on the comments on our manuscript by two enormous reviewers. The correction/clarification table as follows:

**Sl. No.**	**Reviewers' comments**	**Correction/Clarification**
Line number	Comments	Clarification/ explanation
**Reviewer-1**		
1-3	Barcoding of Fish and Shellfish of the world's largest mangrove ecosystem	We shortened the title modifying as **'DNA Barcoding of Brackish and Marine Water Fishes and Shellfishes of Sundarbans, the World's Largest Mangrove Ecosystem.'** We think the suggested title does not clear the theme of the paper. Further, Reviewer-2 did not suggest to edit the original title.
24	Aquatic fauna, be specific looks like fish, bivalves and crabs?	To make more clear, we changed the sentence as "The present study aims to apply a DNA barcoding tool through amplifying two mitochondrial candidate genes i.e., COI and 16S rRNA for accurate identification of fish, aquatic molluscs and crustaceans of Sundarbans mangrove wetland, to build a reference library fish and shellfishes of this unique ecosystems."
44	delete wild animals	deleted 'wild animals' but added 'mammals'
43-63	reduce text by 40%	We did not reduce exactly 40%. However, we have reduced about 25% of the suggested text. More deletion will hamper the chronology and arrangement of understanding the background and importance of the study.
64-78	reduce 40%	
79-98	reduce by 40% is 16s CO1 an issue of debate for your groups?	
99-107	move up with story on mangroves and reduce 40%	
185-188	Numbers do not add up	We described the distribution of sequences numbers of fishes for two genes separately.
189 to 190	you said 3 of each kind earlier—need to show data on numbers of samples somewhere.	To make it clear, we rephrased the statement of specimen collection in Materials and methods section as "At least three individuals were tried to collect for each species. However,

		single individual was also collected for rare species."
Overall comments	Samples of fish, crabs, shrimp, some mollusks were CO1 barcoded and some mitochondrial 16s ribosomal DNA was all sequenced for some. The data for fish seem good, crabs and shrimp ok and mollusks poor. The data are valuable for knowing what fish and crustaceans are present.	Authors want to pay thanks to reviewer for this comment. Actually this is a first initiative to make DNA barcoding of aquatic species of Sundarbans. Complete DNA barcoding of this biome is difficult task. Long time and substantial funding are needed for such work. As an initial work, we believe the data is quite rich for publication. For mollusk, we stated inside paper (Result section) that we failed to amplify the barcode gene for most of the species. That's why number is fewer for molluscs. We also discussed this issue in Discussion part.
	A lot of data are presented (GC content and transitions vs transversions for example) but why they are presented is not well explained.<br>What was actually done is hard to figure out because there has been no filtering. For example, 3 samples were theoretically analyzed for each morphological species but then it turns out one, two 3 or more individual/ individuals were collected and there is no accounting for how many out of the number sampled were successfully sequenced.	Data were tried to explained as per reviewer suggestion. We stated in the Materials and methods section that we tried to collect at least three samples for each species (sometimes more e.g. *Scylla* sp.). However, we collected single individual for rare species. We collected multiple specimen for a species in order to confirm the species by morphological study. However, DNA barcoding is costly in our country, that's why we could not sequence all of the collected specimens. We represented and analyzed those barcode sequences in the article which were found without errors. We did not consider the rate of barcoding success among the number of samples and did not mention in the paper.
	The crab/shrimp data show 11 *Scylla olivivacea* were sequenced out of the 35 total sequences. There are data for 4 species of mollusks. the mollusk data are presented and not discussed	Accurate identification of mud crab (*Scylla sp.*) of Sundarbans was a controversial issue (i.e. which species is dominant, *Scylla serrata* or *S. olivacea*?). Therefore, we collected randomly more (i.e. 11) individuals and barcoded for confirmation of the major species available in this ecosystem and resolved this issue here. For poor numbers of molluscs

		sequence, we clarified it in a previous comments and in the paper (Results and Discussion sections) as well.
	Overall, the paper does not stay on track. The existing introductory sections need to be shortened and the general audience need to be introduced to additional topics (GC content - Tranversions vs transitions-divergence) be explained. The authors address COI vs 16s in the introduction, but then do not return to it in the discussion.	We tried to address all of the comments of reviewer in the revised version. We believe the write up of the paper is okay now. We shortened the Introduction section as mentioned it in an earlier.<br>We added some explanation (importance/ojective) of the analyses of transversion & transition, divergence, GC content in Discussion section of the revised version.<br>In our study, our objective was not to assess or compare which marker i.e. COI or 16S rRNA gene is better as DNA barcode. Rather, we used 16S rRNA as an additional/supplementary tool for those sequences where COI did not give good result. That's why we did not describe this type issue in the Discussion section.
	Having data from this region is important and the data should be presented in a clear concise fashion. I didn't suggest reject because the data are important. The paper should be totally redone.	We agree with reviewer that DNA barcode data for Sundarbans' biome is new for this region. We tried to clarify and make corrections all of the comments of both reviewers.<br>Hope now the paper will meet the reviewers' expectations.
**Reviewer-2**		
Abstract, line 22	Change "apply DNA barcoding tool" to "apply a DNA barcoding tool".	Changed
Abstract, line 25	Change "sequences of 16S rRNA gene" to "sequences of the 16S rRNA gene"	Changed
Abstract, line 26	The reference to "3 aquatic taxa" is not very precise. I'd suggest changing "3 aquatic taxa (fish, crustaceans and mollusk)" to "aquatic fish, crustaceans, and molluscs"	Changed as "fish, crustaceans and molluscs".
Abstract	Give the standard deviations for all the average K2P distances reported.	Standard deviation has been added for all K2P distances.
Abstract, lines, 36-37	Change "misidentification of mud crab species of Sundarbans" to	Changed as "misidentification of the mud crab species of the Sundarbans".

	"misidentification of a mud crab species of the Sundarbans".	Here, 'a' will not be appropriated. Rather use of the word 'the' is more appropriate since mud crab denotes only a particular and single group of crab.
Abstract, line, 37	Change "In case of molluscs" to "In the case of molluscs"	Corrected
Abstract, lines, 38-40	Change last sentence to "The present study describes the development of a molecular and morphometric cross-referenced inventory of the fish and shellfish of the Sundarbans. This inventory will be useful in future biodiversity studies and in forming future conservation plans.	Changed.
Introduction, line, 56	I think instead of "exclusively adapted" you mean "uniquely adapted".	Changed as "uniquely adapted".
Introduction, line, 64	Change "The Sundarbans is the world's single largest continuous mangrove forest lies…" to "The Sundarbans, the world's single largest continuous mangrove forest, lies…"	Changed
Introduction, line, 76	Change "All of these factors educe urgent need…" to "All of these factors highlight the urgent need…"	Changed
Introduction, line, 80	Change "under the term's DNA barcoding or DNA" to "under the terms DNA barcoding or DNA taxonomy"	Changed
Materials and Methods, lines, 151-153	Please give final concentrations of PCR components rather than volumes.	We added PCR concentration.
Materials and Methods, lines, 157	Do you mean a 1.0 % agarose gel?	Yes
Materials and Methods, lines, 168	Was there any reciprocal BLAST or other error checking of the database matches?	Yes. We added "and reciprocal BLAST through NCBI for more confirmation."
Results, line 222:	Delete "respective", it is not needed here.	Deleted "respective"
Results, lines 268-269:	Were the mollusk samples tested for PCR inhibition? If the samples were inhibited, a second DNA	Thanks for the suggestion to reviewer. We will try in our next study.

	extraction, or 10-fold dilution of the DNA sample might improve the sequencing results.	
Discussion, line 283:	Delete “among entities within species”	Deleted.
Discussion, line 284:	Change “runs” to “provides”	Changed as “provides”
Overall comments	The authors produced an ambitious inventory of DNA barcodes of aquatic life in the Sundarbans mangrove ecosystem. They also compared the performance of COI and 16S barcodes. The data will be useful for future biodiversity studies, development of environmental DNA assays, and taxonomy studies. The paper is well-written overall. I recommend publication with no required major revisions.	Authors would like to pay thanks and acknowledge the reviewer for the comments. We also hope this study will be useful for future biodiversity studies, development of environmental DNA assays, and taxonomy studies.
